# Epigallocatechin-3-Gallate Inhibits Stem-Like Inflammatory Breast Cancer Cells

**DOI:** 10.1371/journal.pone.0073464

**Published:** 2013-09-11

**Authors:** Nora D. Mineva, K. Eric Paulson, Stephen P. Naber, Amy S. Yee, Gail E. Sonenshein

**Affiliations:** 1 Department of Biochemistry, Tufts University School of Medicine, Boston, Massachusetts, United States of America; 2 Department of Pathology and Laboratory Medicine, Tufts Medical Center, Boston, Massachusetts, United States of America; University of South Alabama Mitchell Cancer Institute, United States of America

## Abstract

Inflammatory Breast Cancer (IBC) is a highly aggressive form of cancer characterized by high rates of proliferation, lymphangiogenesis and metastasis, and an overall poor survival. As regular green tea consumption has been associated with improved prognosis of breast cancer patients, including decreased risk of recurrence, here the effects of the green tea polyphenol epigallocatechin-3-gallate (EGCG) were tested on two IBC lines: SUM-149 and SUM-190. EGCG decreased expression of genes that promote proliferation, migration, invasion, and survival. Consistently, growth, invasive properties, and survival of IBC cells were reduced by EGCG treatment. EGCG also reduced lymphangiogenesis-promoting genes, in particular *VEGF-D.* Conditioned media from EGCG-treated IBC cells displayed decreased VEGF-D secretion and reduced ability to promote lymphangiogenesis *in vitro* as measured by hTERT-HDLEC lymphatic endothelial cell migration and tube formation. Tumorsphere formation by SUM-149 cells was robustly inhibited by EGCG, suggesting effects on self-renewal ability. Stem-like SUM-149 cells with high aldehyde dehydrogenase (ALDH) activity, previously implicated in poor patient prognosis, were isolated. EGCG treatment reduced growth and induced apoptosis of the stem-like SUM-149 cells in culture. In an orthotopic mouse model, EGCG decreased growth of pre-existing tumors derived from ALDH-positive stem-like SUM-149 cells and their expression of VEGF-D, which correlated with a significant decrease in peritumoral lymphatic vessel density. Thus, EGCG inhibits the overall aggressive IBC phenotype. Reduction of the stem-like cell compartment by EGCG may explain the decreased risk of breast cancer recurrence among green tea drinkers. Recent clinical trials demonstrate the efficacy of green tea polyphenol extracts in treatment of prostate cancer and lymphocytic leukemia with low toxicity. Given the poor prognosis of IBC patients, our findings suggest further exploration of EGCG or green tea in combinatorial treatments against active IBC disease or in maintenance regimens to avoid recurrence is warranted.

## Introduction

Inflammatory breast cancer (IBC) accounts for 1–5% of newly diagnosed breast cancer cases each year in the United States [1]. It is highly aggressive and frequently locally advanced or metastasized at the time of diagnosis [2]. IBC patients often present with a breast that looks inflamed due to extensive lymphovascular invasion of tumor emboli which block lymphatic drainage from the breast, but no palpable tumor [3], [4]. The rapid development of metastases with IBC results from high proliferative rates and potent ability for angiogenesis and lymphangiogenesis [5], [6]. While surgery, radiation and chemotherapy have significantly improved patient prognosis, the outcome remains poor; the 5-year incidence of recurrence is 64.8% compared to 43.4% for patients with similarly staged non-IBC, and the 5-year survival rate is only 40.5% versus 63.2% for non-IBC patients [7]. While no uniform molecular signature currently exists for IBC cells, enrichment of several factors has been reported. For example, E-cadherin has been detected by immunostaining in all inflammatory breast cancer tumors [8], and implicated in the formation of IBC tumor emboli and lymphovascular invasion [8], [9]. Overexpression of RhoC GTPase correlated with the IBC phenotype when compared to similarly staged non-IBC samples by in situ hybridization [10] and has been implicated in IBC cell motility [10], [11]. Similarly, using real-time RT-PCR, Van der Auwera and colleagues demonstrated a significant increase in *VEGF-C* and *VEGF-D* mRNA expression in IBC tumors versus non-IBC samples [12]. VEGF-C and VEGF-D are major lymphangiogenic secretory factors, which have been found to promote lymphatic invasion and metastatic spread of cancer cells [13], [14]. Recently, aldehyde dehydrogenase (ALDH) enzymatic activity has been used to isolate breast cancer cells characterized by enhanced tumorigenicity and self-renewal capacity (stem-like cells) [15]. Consistently, the metastatic aggressive behavior of IBC cells has been attributed to a stem-like cancer cell compartment with high ALDH activity (ALDH-positive cells) [16].

Dietary and environmental exposures play substantial roles in the development of breast cancer. Epidemiological studies have shown that Asian women migrating to the United States dramatically increase their lifetime risk of developing breast cancer and mortality from breast cancer [17], [18]. A comparison of the typical Asian and Western diets revealed, among other things, that the Asian population consumes more green tea. Consumption of green tea has been associated with improved prognosis of patients with breast cancer [19], and regular green tea consumption prior to breast cancer diagnosis is associated with decreased subsequent risk of recurrence [20]. Polyphenols make up approximately 40% of the dry weight of green tea leaves, and include epigallocatechin-3 gallate (EGCG), a compound with significant anti-cancer qualities [21]. As current treatment modalities for IBC are inadequate, here we tested for the first time the effects of EGCG on the distinct growth and dissemination properties of IBC cells in culture and on tumor growth in an orthotopic mouse model. EGCG treatment reduced growth, invasive phenotype, and survival of SUM-149 and SUM-190 IBC cells in culture and their ability to stimulate *in vitro* lymphangiogenesis. Importantly, EGCG reduced the volume, burden and lymphangiogenic potential of pre-established tumors derived from ALDH-positive stem-like SUM-149 cells.

## Materials and Methods

### Ethics Statement

All animal procedures were conducted under an approved protocol #B2012-102 by the Tufts University Institutional Animal Care and Use Committee in accordance with the principals and procedures outlined in the *NIH Guidelines for the Care and Use of Laboratory Animals*. All surgery was performed under Isoflurane anesthesia and all efforts were made to minimize animal suffering.

### Cell Culture and Treatment Conditions

SUM-149 and SUM-190 cells were isolated from primary inflammatory invasive ductal carcinoma by Stephen Ethier (University of Michigan Medical School, Ann Arbor, MI), who kindly provided the cells [22]. Upon receipt, cells were grown in Ham’s F-12 medium (Mediatech) containing 5% fetal bovine serum (FBS) (Invitrogen), 5 µg/ml insulin (Sigma), 1 µg/ml hydrocortisone (Sigma), 100 units/ml penicillin and 100 units/ml streptomycin (Hyclone). Cells were frozen immediately and fresh aliquots thawed and used approximately every 6 weeks. Studies were performed with cells within 6 passages. Cells were confirmed mycoplasma-free using a PCR-based test (VenorGeM Mycoplasma Detection Kit, Sigma). Cultures were treated with doses of EGCG (LKT Laboratories) of 5, 10, 20, 40, 60, 80 or 160 µg/ml which correspond to 10.9, 21.8, 43.6, 87.3, 130.9, 174.5 and 349.0 µM, respectively or with carrier DMSO equivalent to the highest dose of EGCG used (DMSO = 0 µg/ml EGCG). To study secreted proteins, SUM-149 and SUM-190 cells at 85% confluence were grown overnight in serum-free Ham’s F-12 medium containing 0, 5 or 10 µg/ml EGCG. Conditioned media was concentrated using Amicon Ultracel-10 K centrifugal filters (Millipore). Cells were counted and media corresponding to equal cell numbers/condition were used in subsequent experiments. Human telomerase reverse transcriptase - human dermal lymphatic endothelial cells (hTERT-HDLEC) were kindly provided by Todd Reinhart (University of Pittsburgh, Pittsburgh, PA) and grown in complete EGM-2MV Microvascular Endothelial Cell Growth Medium-2 (Lonza) as described previously [23].

### ALDH-positive Cell Sorting and Culture Conditions

To isolate cells based on ALDH activity, an ALDEFUOR Fluorescence Activated Cell Sorting (FACS)-based assay (Stem Cell Technologies) was used. Briefly, SUM-149 cells were suspended at a density of 10^6^ cells/ml in assay buffer with 1.5 µmol/L bodipyaminoacetaldehyde (BAAA) substrate, which is converted to a fluorescent product in the presence of ALDH enzymatic activity. A control sample containing BAAA and 15 µmol/L diethylaminobenzaldehyde (DEAB) ALDH inhibitor was also prepared. Following incubation at 37°C for 45 minutes, fluorescent cells were sorted using a MoFlo Legacy (Beckman Coulter) with a 488 nm laser and a detection filter of 530/40 nm. Dead cells were excluded based on light scatter and propidium iodide (PI) staining. Sorting gates were set outside of the DEAB population to select for cells with the brightest fluorescence and therefore highest ALDH activity (ALDH-positive). The dimmest 10% of the total population, with the lowest ALDH activity, was designated ALDH-negative. Sorted ALDH-positive SUM-149 cells were cultured in Ham’s F-12 medium containing 20 ng/ml EGF (Sigma), 20 ng/ml basic FGF (Fisher Scientific), 1× B27 (Invitrogen), 4 ng/ml Heparin (Sigma), 5 µg/ml insulin, 1 µg/ml hydrocortisone, 100 units/ml penicillin and 100 units/ml streptomycin in low-attachment plates (Corning).

### RT-PCR

RNA was isolated from cells in culture or frozen tumor tissue and cDNA prepared as described [24]. Expression of *CCND1, RHOC*, *FN1*, *CDH1*, *VIM*, *BCL-XL, VEGF-A*, *VEGF-B*, *VEGF-C*, *VEGF-D*, *NANOG*, *STELLA,* control *GAPDH* and *18S* rRNA was assessed by reverse transcription (RT)-PCR using the thermal cycler conditions and primer sets detailed in Materials S1. Resulting PCR products were subjected to gel electrophoresis and densitometry. Expression levels, normalized to *GAPDH* or *18S* rRNA, are given as fold change relative to experimental control sample set to 1.0 from 3 independent experiments.

### Cell Growth and Survival Analyses

As a measure of cellular metabolism and therefore growth and viability, ATP levels were assessed in 3.0×10^3^ SUM-149 cells and 4.0×10^3^ SUM-190 cells using an ATPlite luminescence ATP detection assay system (Perkin Elmer), as described previously [24]. For comparison of ATP levels in the total SUM-149 cell population *vs* sorted ALDH-positive SUM-149 cells, 1.25×10^3^ cells were used. Average ATP levels, for triplicate samples, are presented as percent of controls ± Standard Deviation (SD). For evaluation of cellular DNA content, cells were treated with the indicated doses of EGCG for 72 h, stained with PI and analyzed by FACS, as previously described [25]. Data (5,000 events) were collected on a linear scale to assess DNA content and analyzed using WinMDI software. For trypan blue exclusion experiments, 5.0×10^3^ unsorted SUM-149 cells or sorted ALDH-positive SUM-149 cells were plated in triplicate in 96-well tissue culture treated or low attachment plates, respectively. Following treatment with the indicated doses of EGCG for 24 h, supernatants and cells (trypsinized and syringed to a single cell suspension) were exposed to 0.4% trypan blue. The average percent of dead cells was calculated as the number of trypan blue positive cells divided by the total number of cells multiplied by 100± SD. Data are shown from a representative of three independent experiments. Caspase-3 and −7 activities were assessed using a Caspase-Glo 3/7 Luminescent Assay (Promega). Briefly, 2.5×10^3^ ALDH-positive SUM-149 cells were plated in triplicate in 96-well low attachment plates and treated with the indicated doses of EGCG for 24 h. Luminescence was measured and values normalized to cell viability (ATP levels from similarly treated samples). Values shown are average fold changes (relative to control set to 1) ± SD of three independent experiments combined.

### Soft Agar and Matrigel Assays

For soft agar assays, 1.0×10^5^ SUM-149 or 2.5×10^5^ SUM-190 cells in a mix of 0.4% Bacto Agar (BD Biosciences) in complete media with the indicated doses of EGCG were plated on six-well dishes pre-coated with a 1∶1 mix of 2×Ham’s F-12 medium supplemented with 10% FBS and 1.6% Bacto Agar. Cells were fed 3 times/week with complete Ham’s F-12 medium containing DMSO or the indicated dose of EGCG. After 3 weeks, cells were stained overnight with 0.2 mg/ml iodonitrotetrazolium chloride (Sigma) and photographed at 40×magnification. Colonies with diameters of approximately 20 microns or greater were counted using ImageJ software (NIH). Matrigel assays were carried out as described previously [26] using single cell suspensions of 5.0×10^3^ SUM-149 cells or 7.5×10^3^ SUM-190 cells with Ham’s F-12 media containing 0 or 40 µg/ml EGCG. Cultures were incubated for 10 days or 14 days for SUM-149 and SUM-190, respectively and photographed.

### Immunoblot Analysis

Whole cell extracts from cells in culture or frozen homogenized tumor tissue were prepared and immunoblotted as previously described [27]. Antibodies against cleaved Caspase-3 (#9661) and cleaved PARP (#51-9000017) were from Cell Signaling and BD Biosciences, respectively. Mouse monoclonal anti-human VEGF-D (MAB622) and β-actin antibodies were from R&D Systems and Sigma, respectively. To detect secreted VEGF-D protein, concentrated conditioned media corresponding to 1.0×10^6^ IBC cells/condition were used in immunoblotting.

### Wound Healing Assays

Cultures of hTERT-HDLEC cells at 95% confluence were scratched with a sterile 200 µl micropipette tip, washed with 1× PBS and exposed to EBM-2 Basal Medium (Lonza) supplemented with 1% FBS and concentrated conditioned Ham’s F-12 medium (corresponding to 1.0×10^6^ SUM-149 or 1.5×10^6^ SUM-190 cells treated with DMSO or EGCG for 24 h). Alternatively, Ham’s F-12 medium incubated with either DMSO or EGCG in the absence of IBC cells was concentrated and used. The same fields of the wound margin were photographed at 100×magnification at 0 and 24 h. Wound areas were determined using ImageJ software. Values for percent wound closure were calculated for each condition by subtracting the remaining (unclosed) wound area at 24 h from the wounded area at 0 h (set to 100%). Values shown are averages of triplicate samples ± SD.

### Tube Formation Assays

Tube formation assays were performed as described [28]. hTERT-HDLEC (1.5×10^4^) cells were plated, in triplicate, on glass chambers coated with growth factor reduced Matrigel (BD Biosciences). Cells were incubated in 200 µl EBM-2 Basal Medium supplemented with 1% FBS and concentrated conditioned medium from DMSO- or EGCG-treated SUM-149 or SUM-190 cells (volume corresponding to 0.65×10^6^ or 1.0×10^6^ cells, respectively). Alternatively, concentrated DMSO- or EGCG-treated control Ham’s F-12 medium (no IBC cells) was added. Following incubation for 13 h, three random fields/well were photographed (40×magnification). Incomplete networks were excluded and numbers of closed networks of vessel-like tubes counted in 3 fields (n = 9). The average percentage relative to control samples is presented ± SD.

### VEGF-D Immunodepletion

VEGF-D was immunodepleted from conditioned concentrated medium from untreated SUM-149 or SUM-190 cells using a rabbit anti-VEGF-D antibody (sc-25784) or control normal rabbit IgG (sc-2027) [both from Santa Cruz Biotechnology] bound to rec-Protein G-Sepharose 4B beads (Invitrogen), or alternatively crosslinked with bis(sulfosuccinimidyl) suberate (Thermo Scientific) to Protein G Dynabeads (Invitrogen) as recommended by the manufacturer. Immunodepleted medium was used in wound healing or tube formation assays or immunoblotted for VEGF-D.

### Tumorsphere Formation Assay

Single cell suspensions of 2.0×10^4^ SUM-149 cells in complete Ham’s F-12 medium containing the indicated concentrations of EGCG were plated, in triplicate, on low attachment six-well dishes. After 7 days of culture, primary spheres were photographed at 200×magnification and those with diameters of ∼125 microns or greater counted manually using the objective ruler. Primary spheres in control DMSO treated group were then collected by centrifugation, digested using Accutase (Invitrogen) and 5×10^3^ single cells subcultured for secondary sphere formation in the presence of EGCG. For tertiary sphere formation, 10^3^ cells obtained from DMSO-treated, digested secondary spheres were used. Culture conditions and analysis of secondary and tertiary spheroids was performed as for primary spheres. Sphere forming efficiency was calculated as number of spheres formed/total number of cells plated×100. Values shown are averages ± SD of three independent experiments combined.

### Xenograft Mouse Model

Six-week-old female nonobese diabetic/severe combined immunodeficient (NOD/SCID) mice (Jackson Laboratory) were implanted with 5.0×10^3^ sorted ALDH-positive SUM-149 cells in a 30 µL 50% Matrigel (BD Biosciences, CB-40230A) solution (1∶1 dilution of Matrigel with Ham’s F-12 medium) in the fourth inguinal mammary fat pad. Once palpable tumors were detected, mice were separated into two groups (n = 6), weighed, and administered a 0.1 ml intraperitoneal injection of 16.5 mg/kg EGCG (Sigma, E4143) or control PBS five times a week for the first five weeks and daily for the last week. Tumor size was measured with calipers twice a week and tumor volumes were calculated using the formula: (length × width^2^)/2. Mice were given autoclaved food and water ad libitum until sacrifice. Tumors were dissected, weighed, sectioned and snap frozen in liquid nitrogen for RNA and protein isolation or fixed in 10% Neutral Buffered Formalin for histological analysis.

### Immunohistochemistry and Evaluation of Lymphatic Vessel Density

For evaluation of lymphatic vessel density, fixed tumor tissues were paraffin embedded and 4-µm-thick sections prepared using standard procedures at the Tufts University Division of Laboratory Animal Medicine Histology Core. For each mouse, 4 slides/tumor were cut 200 µm apart and subjected to immunostaining for podoplanin. Briefly, tumor sections were deparaffinized, rehydrated through a descending ethanol gradient and subjected to heat-induced antigen retrieval. Sections were blocked with Renaissance Background Reducing Diluent (Biocare Medical) for 30 minutes and stained with anti-podoplanin hamster monoclonal antibody 8.1.1 (Developmental Studies Hybridoma Bank, University of Iowa, IA) at a dilution of 1∶500 overnight at 4°C. Slides were stained using a Benchmark XT instrument (Ventana Medical Systems) at the Tufts Medical Center Department of Pathology and Laboratory Medicine. Slides were examined at low magnification and three areas with the greatest numbers of vessels (hot spots) at the tumor periphery/peritumoral area (defined as a 2-mm-wide band containing the invasive front) were identified for each tumor slice. These were photographed at 400×magnification and the numbers of vessels/hotspot counted and the average value of Vessel Density/slide plotted. If no hot spots were detected, these tumor sections were given a zero count. One tumor section from the Control group displayed less than 5% peripheral tumor tissue and was excluded from analysis. Thus, we compared n = 24 for the EGCG-treated *vs* n = 23 for the Control group.

### Statistical Analysis

Statistical significance between samples was determined using a one-way ANOVA with a Tukey HSD post hoc test for wound healing and tube formation assays using conditioned media from IBC cells treated with EGCG or DMSO, or control conditioned media. All other analyses were performed using a two-tailed Student’s *t*-test for samples with equal variance.

## Results

### EGCG Inhibits Expression of Genes that Promote Growth, Transformed Phenotype and Survival of IBC Cells

**Figure 1:**
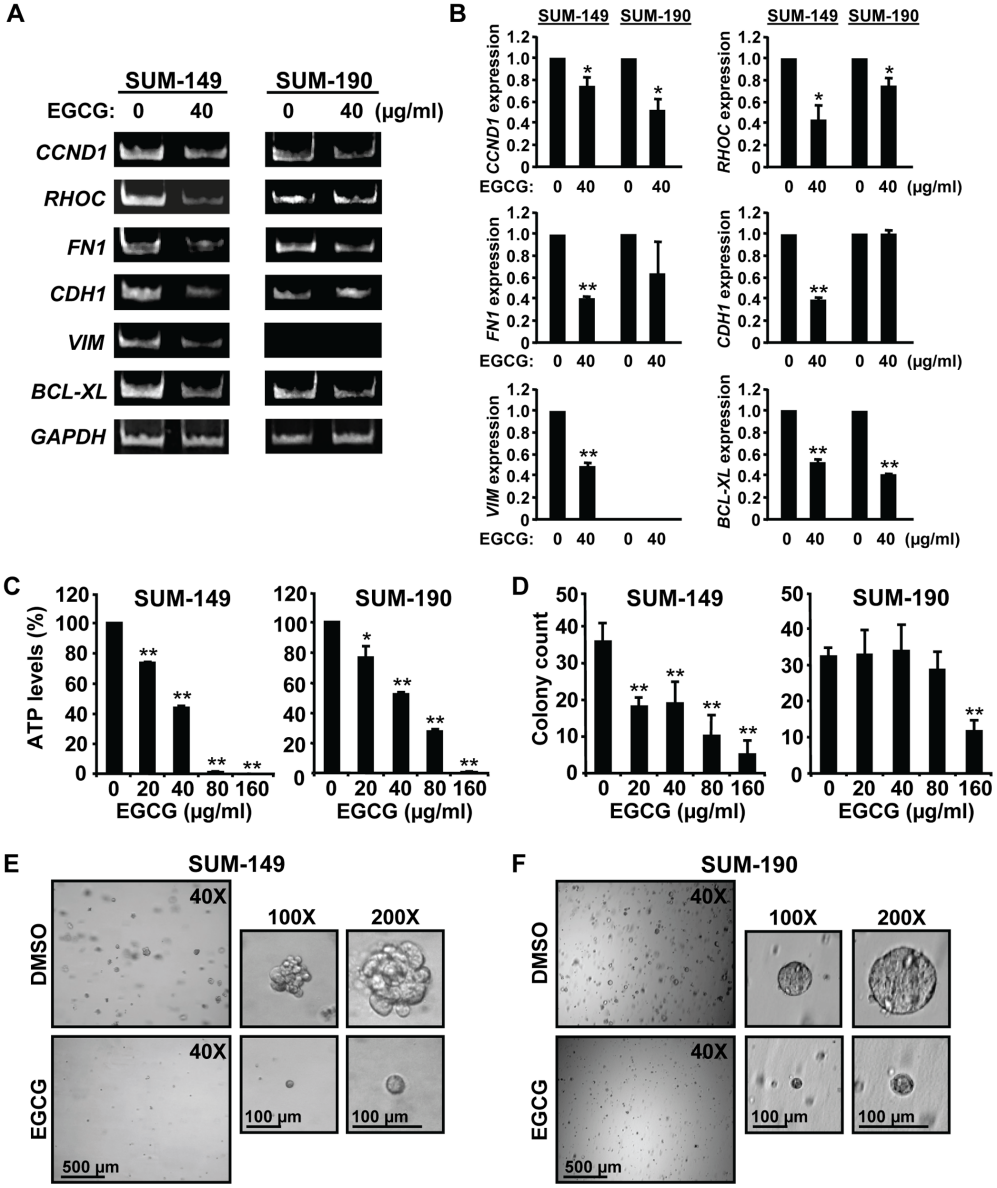
EGCG alters gene expression, growth and invasive phenotype of IBC cells. (A–B) RNA analysis. SUM-149 (left panels) and SUM-190 cells (right panels) were treated with 40 µg/ml EGCG or equivalent volume of carrier DMSO (0 µg/ml EGCG) for 72 h. RNA was analyzed by RT-PCR for levels of *Cyclin D1* (*CCND1*), *RHOC*, *Fibronectin* (*FN1*), *E-cadherin* (*CDH1*), *Vimentin* (*VIM*) and *BCL-XL* and representative images shown (A). Values for RNA expression were obtained from 3 independent experiments by densitometry and normalization to the loading control *GAPDH*, and are given as average fold changes ± SD relative to experimental control sample set to 1.0. (B). *, p-value <0.05; **, p-value <0.005. (C) ATP levels. SUM-149 (left panel) and SUM-190 cells (right panel) were treated, in triplicate, with the indicated doses of EGCG for 72 h. ATP levels were measured and analyzed as described in Materials and Methods. *, p-value <0.05 and **, p-value <0.005 for comparison of individual EGCG doses to control untreated sample. (D) Anchorage independent growth. SUM-149 (left panel) and SUM-190 cells (right panel) were subjected to a soft agar colony assay using the indicated concentrations of EGCG. Colonies were counted and averages of triplicate samples ± SD are presented. **, p-value <0.005 for comparison of individual EGCG doses to control untreated sample. (E–F) Matrigel outgrowth. SUM-149 (E) and SUM-190 cells (F) were subjected to a Matrigel invasion assay in medium containing 0 or 40 µg/ml EGCG for 10 days or 14 days for SUM-149 and SUM-190, respectively. Representative images are shown at the indicated magnifications.

As an initial test of the effects of EGCG on IBC cells, a preliminary dose-response curve was performed on SUM-149 and SUM-190 cells and a concentration of 40 µg/ml EGCG selected, which inhibited growth of both lines by ∼50% (not shown and see Fig. 1C). Using this dose, the effects of EGCG on RNA expression of genes that control proliferation, migration, invasion, and survival were examined (Fig. 1A). The data from this and two additional independent experiments were quantified, normalized to the *GAPDH* control and averages ± SD presented in Figure 1B. EGCG substantially decreased mRNA levels of *CCND1*, encoding the proliferation marker Cyclin D1, *RHOC* and *FN1* in both lines; although the decrease in *FN1* did not reach statistical significance for SUM-190 cells. RhoC and fibronectin have been implicated in IBC signaling and breast cancer cell migration [10], [11], [29]. IBC cells typically overexpress E-cadherin, which promotes their ability to form emboli [8], [9]. EGCG reduced RNA levels of *CDH1,* encoding E-cadherin, in SUM-149 but not in SUM-190 cells. RNA expression of the mesenchymal marker *VIM* was reduced by EGCG in SUM-149 cells, but was not detectable in SUM-190 cells (Fig. 1A). Lastly, EGCG reduced expression of the pro-survival *BCL-XL* gene in both lines. Thus, EGCG inhibits the expression of genes that promote IBC cell growth, transformed phenotype and survival, and these properties are explored below.

### EGCG Reduces IBC Cell Growth, Invasive Phenotype and Survival

As a measure of cellular metabolism and therefore growth and viability, ATP levels were assessed. EGCG treatment caused a robust, dose-dependent decrease in ATP levels in SUM-149 and SUM-190 cells (Fig. 1C), which was more profound in SUM-149 cells at the higher doses. To study anchorage-independent growth, cells were grown in soft agar (Fig. 1D). The number of SUM-149 colonies formed was significantly decreased with EGCG in a dose-dependent manner. SUM-190 colony numbers were only decreased at 160 µg/ml EGCG. We next examined invasive outgrowth in Matrigel (Fig. 1E and 1F). Both SUM-149 and SUM-190 cells formed spheres, which resembled tumor emboli typically seen infiltrating the dermal lymphatic channels of IBC patients [8]. Within 10 days of culturing, SUM-149 cells formed large multi-focal 3D spheroids with “grape-like” morphology [30]. Treatment with 40 µg/ml EGCG robustly reduced the size and invasive characteristics of these spheroids (Fig. 1E). SUM-190 cells required a somewhat longer incubation time (14 days) to form similarly sized spheres, which had a round outline, resembling “mass” morphology [30] (Fig. 1F). EGCG treatment resulted in a substantial reduction in the size of SUM-190 spheres.

**Figure 2:**
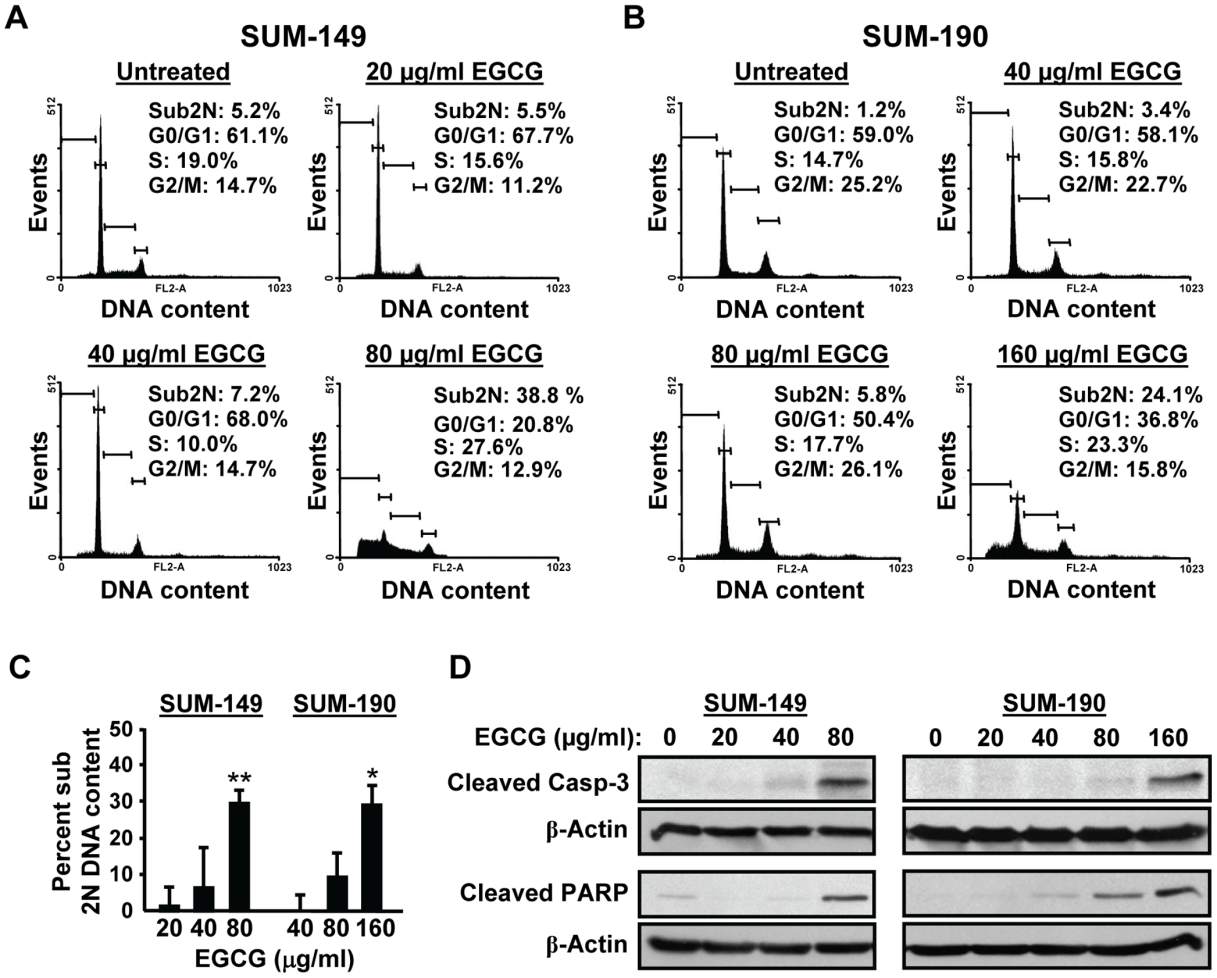
Higher doses of EGCG reduce survival of IBC cell lines. (A) SUM-149 and (B) SUM-190 cells were treated with the indicated doses of EGCG for 72 h and subjected to FACS analysis. Data from one representative experiment are shown. (C) Three independent FACS analyses were performed as in parts A and B. Percent sub 2N DNA content in each treatment group is presented as an average of the three analyses ± SD. *, p-value <0.05; **, p-value <0.005 for comparison to control untreated sample. (D) Whole cell extracts (50 µg) isolated from SUM-149 (left panels) and SUM-190 cells (right panels) were subjected to immunoblot analysis for cleaved Caspase-3 (Casp-3), cleaved PARP and β-actin, as a loading control. Data from one representative of 3 independent experiments are shown.

To test whether EGCG induces apoptosis, DNA content was quantified by FACS analysis. A substantial increase in the percentage of cells with a sub-2N DNA content was detected in SUM-149 cells with 80 µg/ml EGCG (Fig. 2A), and no viable cells remained with 160 µg/ml EGCG (data not shown). SUM-190 cells were more resistant, and a significant extent of cell death was seen only at 160 µg/ml EGCG (Fig. 2B). Results from three independent experiments confirm that ∼30% of SUM-149 cells undergo apoptosis at 80 µg/ml EGCG, whereas a higher EGCG concentration of 160 µg/ml is needed to see the same extent of SUM-190 cell death (Fig. 2C). Cleavage of Caspase-3 and PARP proteins was also monitored. Consistently, levels of cleaved Caspase-3 and PARP were significantly elevated in SUM-149 and SUM-190 cells at 80 and 160 µg/ml of EGCG, respectively (Fig. 2D). In summary, EGCG causes a dose-dependent decrease in growth of SUM-149 and SUM-190 IBC cells at lower concentrations and at higher doses induces apoptosis. The ability of the IBC cells to form spheres in Matrigel is also greatly reduced by EGCG treatment.

### EGCG Treatment of IBC Cells Reduces Secretion of Factors that Stimulate *in vitro* Lymphangiogenesis

**Figure 3:**
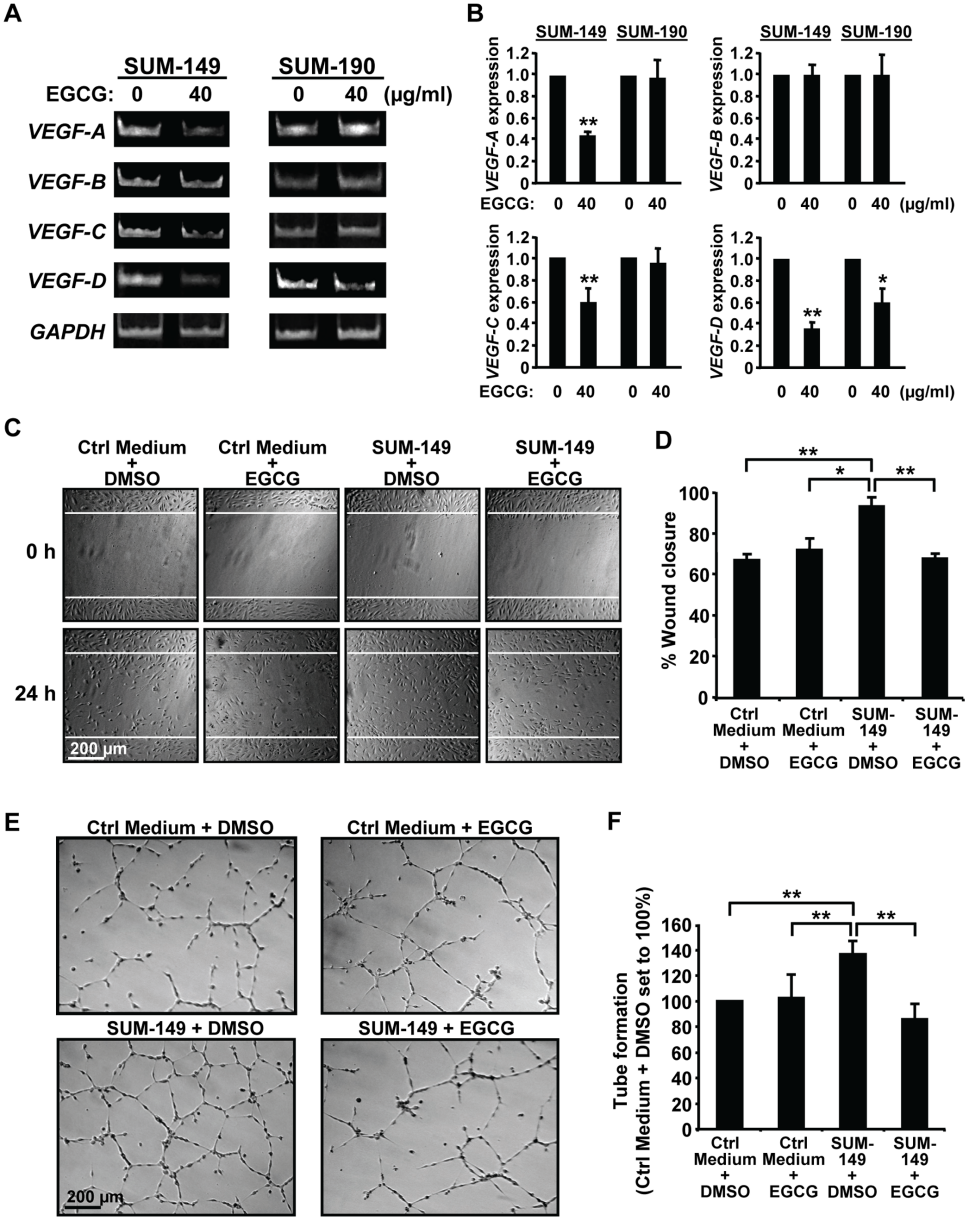
EGCG prevents stimulation of lymphatic endothelial cell migration and tube formation by SUM-149 cells. (A–B) RNA analysis. SUM-149 (left panels) and SUM-190 cells (right panels) were treated with 40 µg/ml EGCG or DMSO (0 µg/ml EGCG) for 72 h. RNA was analyzed by RT-PCR for levels of *VEGF-A*, *VEGF-B*, *VEGF-C*, *VEGF-D* and *GAPDH*. (A) Images from a representative experiment are shown. (B) Values for RNA expression from three independent experiments were normalized to the loading control *GAPDH* and are given as average fold change ± SD relative to the experimental control samples set to 1.0. *, p-value <0.05; **, p-value <0.005. (C–D) Wound healing assays. Scratched hTERT-HDLEC cultures were exposed to EBM-2 Basal Medium plus 1% FBS and supplemented with concentrated Ham’s F-12 medium from SUM-149 cells treated with DMSO (0 µg/ml EGCG) (SUM-149+ DMSO) or 10 µg/ml EGCG (SUM-149+ EGCG) corresponding to equal cell numbers/condition. Alternatively, similarly treated concentrated conditioned Ham’s F-12 medium from plates without cells was used (Control [Ctrl] medium). Wound scratches were photographed and analyzed as described in Materials and Methods. White lines indicate the position of the original scratch. Representative images are shown (C). Percent wound closure values represent averages of triplicate samples ± SD (D). *, p-value <0.05; **, p-value <0.005. (E–F) Tube formation assays. hTERT-HDLEC cells were subjected to tube formation assays as described in Materials and Methods using EBM-2 Medium supplemented as in part C. Representative images are shown (E). Values for closed networks are given as averages of nine fields ± SD (F). **, p-value <0.005.

**Figure 4:**
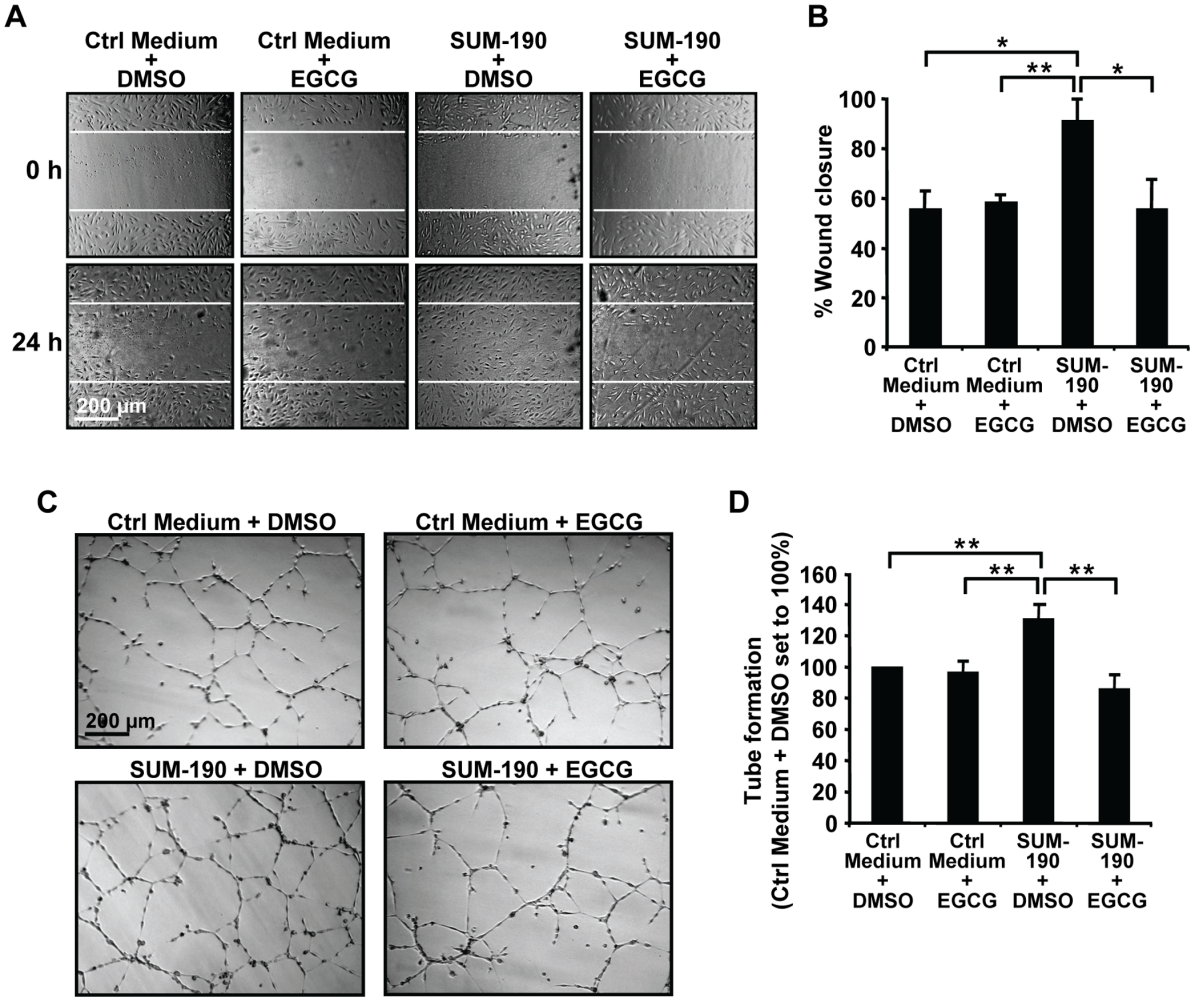
EGCG prevents stimulation of lymphatic endothelial cell migration and tube formation by SUM-190 cells. (A–B) Wound healing assays of the effects of EGCG on secretion of factors by SUM-190 cells were performed as described above for SUM-149 cells in Fig. 3C and 3D. White lines indicate the position of the original scratch. Representative images are shown (A). Percent wound closure values shown are averages of triplicate samples ± SD (B). *, p-value <0.05; **, p-value <0.005. (C–D) hTERT-HDLEC cells were subjected to tube formation assays with SUM-190 cell conditioned media as in Fig. 3E and 3F. Representative images are shown (C). Values for closed networks are given as averages of nine fields ± SD (D). **, p-value <0.005.

Since growth and dissemination of IBC tumors require angiogenesis and lymphangiogenesis, which are mediated by VEGF family members VEGF-A, VEGF-B, VEGF-C, and VEGF-D [31], the effects of EGCG on RNA expression of these genes was assessed next (Fig. 3A and 3B). EGCG treatment of SUM-149 cells significantly decreased *VEGF-A*, *VEGF-C* and *VEGF-D* RNA levels while *VEGF-B* expression was unaffected. In SUM-190 cells, only a decrease in *VEGF-D* levels was seen with EGCG. Notably, *VEGF-D* is significantly elevated in primary IBC samples compared to non-IBC tumors [12], and implicated in promoting tumor dissemination by lymphangiogenesis [31]. The ability of IBC cells to secrete factors that stimulate lymphangiogenesis, and the effects of EGCG were next assessed by using lymphatic endothelial cell migration and tube formation assays. Serum-free medium was used to avoid FBS, which clogs the filters used for concentration. This permits lowering the EGCG dose to 10 µg/ml as the polyphenol is more stable in the absence of serum factors. Conditioned serum-free media from untreated and EGCG-treated IBC cells or from plates without cells were collected and concentrated. Media without cells showed no effects on hTERT-HDLEC cell migration (Figs. 3C and 3D, 4A and 4B) or survival (data not shown), suggesting EGCG had no direct effects on these cells. Addition of concentrated conditioned medium from DMSO-treated SUM-149 cells (Fig. 3C and 3D) or SUM-190 cells (Fig. 4A and 4B) led to statistically significant increases in wound closure at 24 h. EGCG treatment of SUM-149 or SUM-190 cells prevented this increase, such that percent wound closure was equal to that seen with control media (Fig. 3C and 3D and Fig. 4A and 4B). Thus, secretion of factors by IBC cells that promote migration of hTERT-HDLEC cells is inhibited by EGCG.

We next assessed the effects of conditioned media on tube formation by hTERT-HDLEC cells. Compared to control media, a greater number of closed networks of vessel-like tubes was seen when hTERT-HDLEC cells were incubated with media from SUM-149 (Fig. 3E) or SUM-190 cells (Fig. 4C). Quantification of the images indicated that the differences were significant for both cell lines (Figs. 3F and 4D). Stimulation of tube formation by media from SUM-149 (Fig. 3E and 3F) or SUM-190 (Fig. 4C and 4D) cells was prevented by EGCG; whereas, addition of media containing EGCG incubated in the absence of cells had no effect on the number of tubes formed. Thus, IBC cells secrete factors that stimulate endothelial cell tube formation and EGCG treatment inhibits these effects.

### VEGF-D Secretion by IBC Cells Promotes Lymphatic Endothelial Cell Migration and Tube Formation

**Figure 5:**
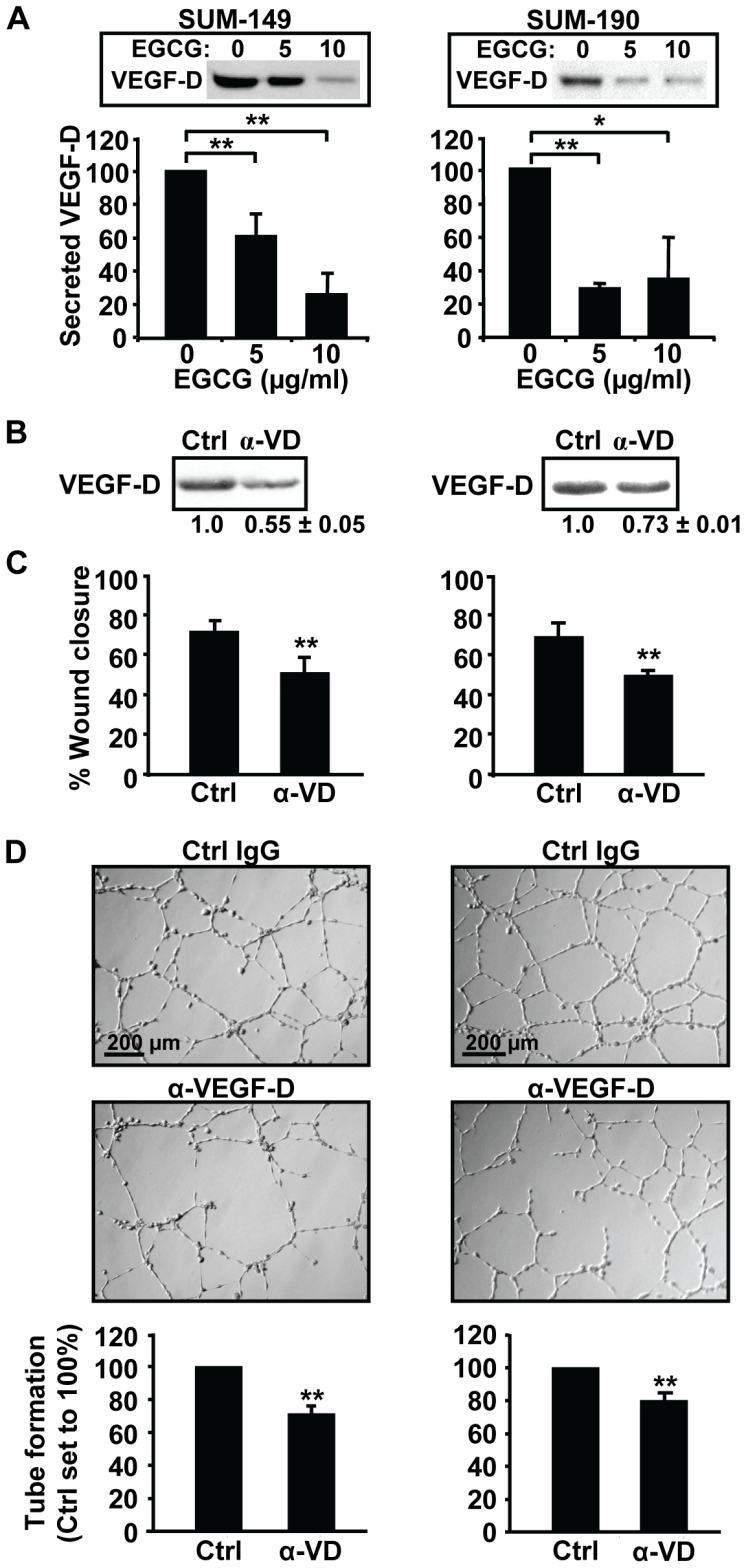
Depletion of VEGF-D reduces stimulation of lymphatic endothelial cell migration and tube formation. (A) SUM-149 (left panel) or SUM-190 cells (right panel) at 85% confluence were grown overnight in medium containing 0, 5 or 10 µg/ml EGCG. Concentrated conditioned media from equal cell numbers were subjected to immunoblotting for VEGF-D (insets). Blots from three separate experiments were scanned and average VEGF-D levels ± SD are given as a percentage relative to untreated cells (set to 100%). *, p-value <0.05; **, p-value <0.005. (B) Conditioned media from untreated SUM-149 (left panel) or SUM-190 cells (right panel) was depleted with a VEGF-D antibody (α-VD) or a control IgG antibody (Ctrl), and then subjected to immunoblotting for VEGF-D. A representative experiment is shown. Average VEGF-D levels ± SD relative to control IgG sample set to 1.0 from 3 independent experiments are given below. (C–D) hTERT-HDLEC cultures were subjected to wound healing (C) or tube formation (D) assays in depleted media from SUM-149 (left panels) or SUM-190 (right panels) cells. A representative of three independent experiments is shown. Percent wound closure was determined as in Fig. 3D and tube formation was quantified as in Fig. 3F. ** p-value <0.005.

VEGF-D has the ability to promote lymphangiogenesis [31]. Given our data showing the green tea polyphenol reduces *VEGF-D* RNA levels, the effects of EGCG on VEGF-D secretion were examined. Cells were incubated with 0, 5 or 10 µg/ml EGCG and VEGF-D levels in the conditioned concentrated media measured. EGCG robustly reduced secretion of VEGF-D protein from both IBC lines (Fig. 5A). To directly test the role of VEGF-D secretion in lymphangiogenic properties of IBC cells, conditioned medium from untreated cells was immunodepleted with an anti-VEGF-D antibody or normal IgG, as a control and then used in wound healing and tube formation assays. Following immunodepletion, VEGF-D levels were decreased by ∼45% and 27% in SUM-149 and SUM-190 media, respectively (Fig. 5B); whereas, levels of VEGF-A and VEGF-C were unaffected (Figure S1), confirming the specificity of the antibody. VEGF-D depletion significantly decreased the ability of SUM-149 and SUM-190 conditioned media to stimulate wound closure by hTERT-HDLEC cells (Fig. 5C). To assess the role of VEGF-D in tube formation assays, we first tested the effects of addition of 50 ng/ml recombinant human VEGF-D protein. VEGF-D addition resulted in an increase in hTERT-HDLEC tube formation of ∼29% (data not shown). Consistently, VEGF-D depletion in SUM-149 and SUM-190 cell conditioned media led to substantially reduced numbers of closed networks of vessel-like tubes (Fig. 5D). Thus, secretion of VEGF-D by IBC cells, which stimulates lymphatic endothelial cell migration and tube formation, is strongly inhibited by EGCG.

### EGCG Inhibits Stem-like SUM-149 Cells in Culture

**Figure 6:**
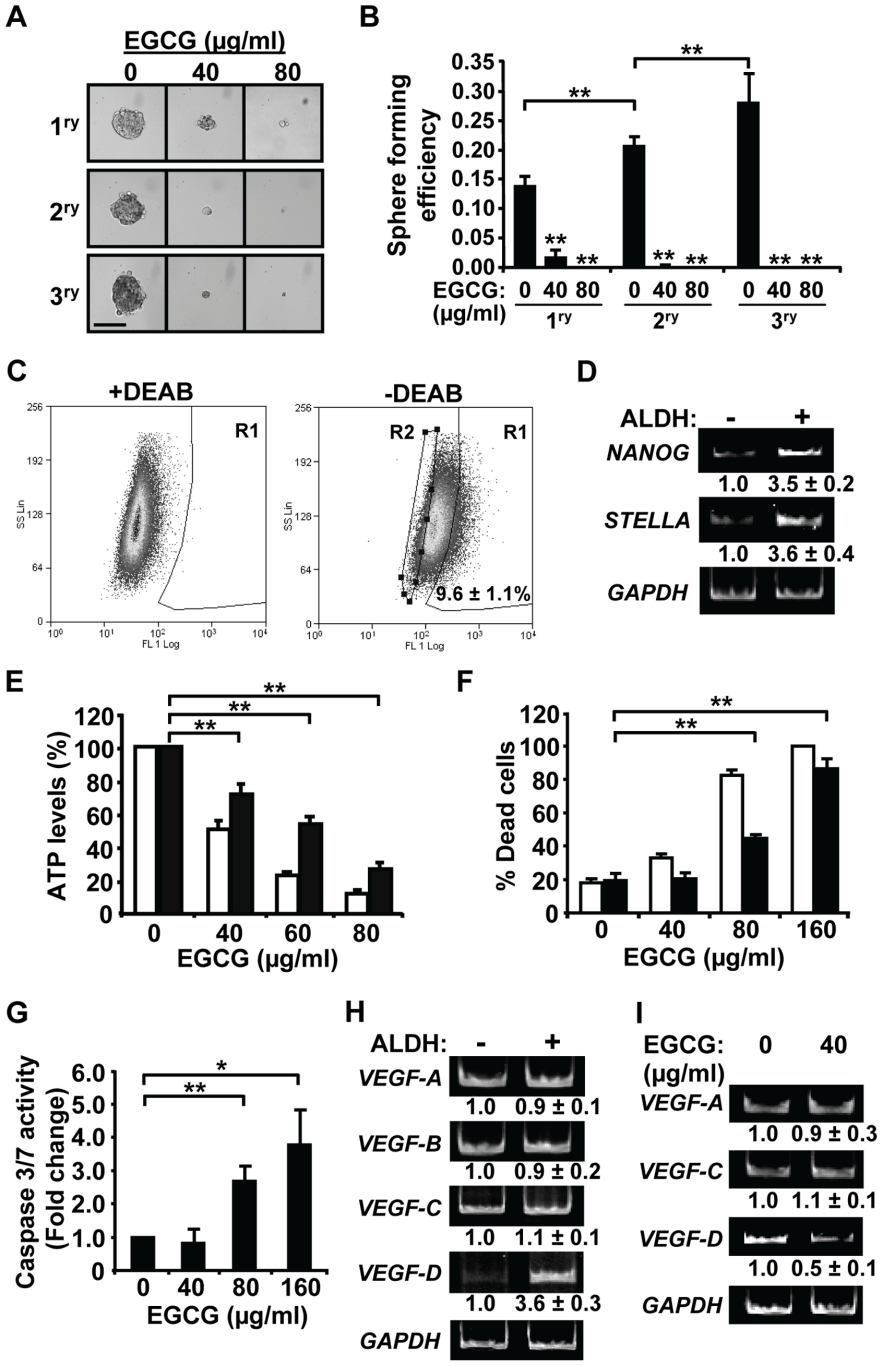
EGCG reduces viability and *VEGF-D* expression of IBC stem-like cells *in vitro*. (A–B) SUM-149 cells were subjected to primary (1^ry^), secondary (2^ry^) and tertiary (3^ry^) tumorsphere formation assays under low attachment conditions in the absence or presence of EGCG. After 7 days of culture, spheres with a diameter ∼125 µm or greater were counted. Representative images are shown (A). Sphere forming efficiency was calculated as number of spheres formed/total number of cells plated×100. Values given represent averages ± SD from three independent experiments (B). Scale bar = 125 µm. **, p-value <0.005 for comparison to control untreated sample or as indicated. (C) ALDH activity. SUM-149 cells were subjected to a FACS-based ALDEFUOR assay. Left and right panels: scatter plots of cells incubated with ALDH substrate in the presence or absence of the ALDH inhibitor DEAB, respectively. R1- ALDH-positive cells; R2- ALDH-negative cells. Percent ALDH-positive cells represents average of three experiments ± SD. (D) RNA from ALDH-positive vs -negative cells was analyzed by RT-PCR and a representative of two independent experiments shown. Values ± Standard Error (SE) are given below for fold change relative to ALDH-negative sample set to 1.0. (E–G) Unsorted SUM-149 cells (white bars) or sorted ALDH-positive SUM-149 cells (black bars) were treated with EGCG for 24 h. ATP levels (E) and percent dead cells (F) were determined as described in Materials and Methods. **, p-value <0.005. (G) Caspase-3/7 activity was measured in ALDH-positive cells using a Caspase-Glo 3/7 assay. *, p-value <0.05; **, p-value <0.005. (H–I) RT-PCR analysis was performed for VEGF family members in ALDH-positive *vs* -negative cells (H) and in ALDH-positive cells treated with EGCG for 24 h (I). A representative experiment in each case is shown. Expression values represent average fold change of three independent experiments ± SD relative to control samples set to 1.0.

Wicha and coworkers have demonstrated the presence of a cancer stem-like cell population in the SUM-149 but not the SUM-190 cell line [32]. As an initial test of the effects of EGCG on SUM-149 cell self-renewal capacity, an *in vitro* tumorsphere assay was performed under low attachment conditions with serial passage (Fig. 6A). Primary SUM-149 tumorsphere formation was potently decreased with 40 µg/ml EGCG and 80 µg/ml EGCG completely inhibited their formation. Dissociation of primary spheres and passage onto secondary and subsequent tertiary spheres led to an increase in sphere forming efficiency at each step consistent with enrichment of cells with self-renewal capacity (Fig. 6B). Notably, formation of secondary and tertiary spheres was almost completely inhibited in the presence of EGCG even at the lower dose of 40 µg/ml (Fig. 6A and 6B).

To specifically test the effects of EGCG on the stem-like cell compartment, ALDH-positive SUM-149 cells were isolated using a FACS-based ALDEFLUOR assay. As noted above, SUM-190 cells are ALDH-negative [32] (data not shown). The SUM-149 cell population contained 9.6±1.1% ALDH-positive cells as judged by comparison to a sample with the ALDH inhibitor DEAB (Fig. 6C, R1 gate). The dimmest 10% of the total population was designated ALDH-negative (Fig. 6C, R2 gate). Consistent with isolation of a stem-like cell compartment, increased expression of pluripotent stem cell markers *NANOG* and *STELLA* was observed in ALDH-positive SUM-149 cells as compared to ALDH-negative ones (Fig. 6D). EGCG treatment of SUM-149 ALDH-positive cells led to a significant dose-dependent decrease in ATP levels, although it was more moderate than that seen for the total SUM-149 cell population (Fig. 6E). Treatment of ALDH-positive SUM-149 cells with the higher EGCG doses (80 and 160 µg/ml) induced cell death (Fig. 6F and 6G); although again to a lesser extent than seen for the total population. These findings are consistent with the notion that the ALDH-negative cells, which represent ∼90% of the total population, are more sensitive to EGCG. ALDH-positive breast cancer cells have been reported to localize to tumor regions with increased microvessel density [33], suggesting potential paracrine interactions with endothelial cells. Thus, we next examined the expression of VEGF family members in ALDH-positive *vs* ALDH-negative SUM-149 cells. Interestingly, RT-PCR analyses revealed that *VEGF-D* expression was substantially higher in ALDH-positive *vs* ALDH-negative cells, whereas both populations had similar levels of *VEGF-A*, *VEGF-B*, and *VEGF-C* RNA (Fig. 6H). Since EGCG treatment reduced the levels of *VEGF-A*, *VEGF-C* and *VEGF-D* in the total SUM-149 cell population, we next assessed the expression of these factors following polyphenol treatment of the purified ALDH-positive cells. Notably, EGCG reduced the levels of *VEGF-D* RNA by ∼50%, while no change was seen in *VEGF-A* or *VEGF-C* RNA levels (Fig. 6I). Thus, EGCG reduces the self-renewal capacity of SUM-149 cells and the growth, *VEGF-D* expression and survival of the ALDH-positive stem-like subpopulation.

### EGCG Reduces Growth of Pre-existing Tumors Derived from SUM-149 Stem-like Cells

**Figure 7:**
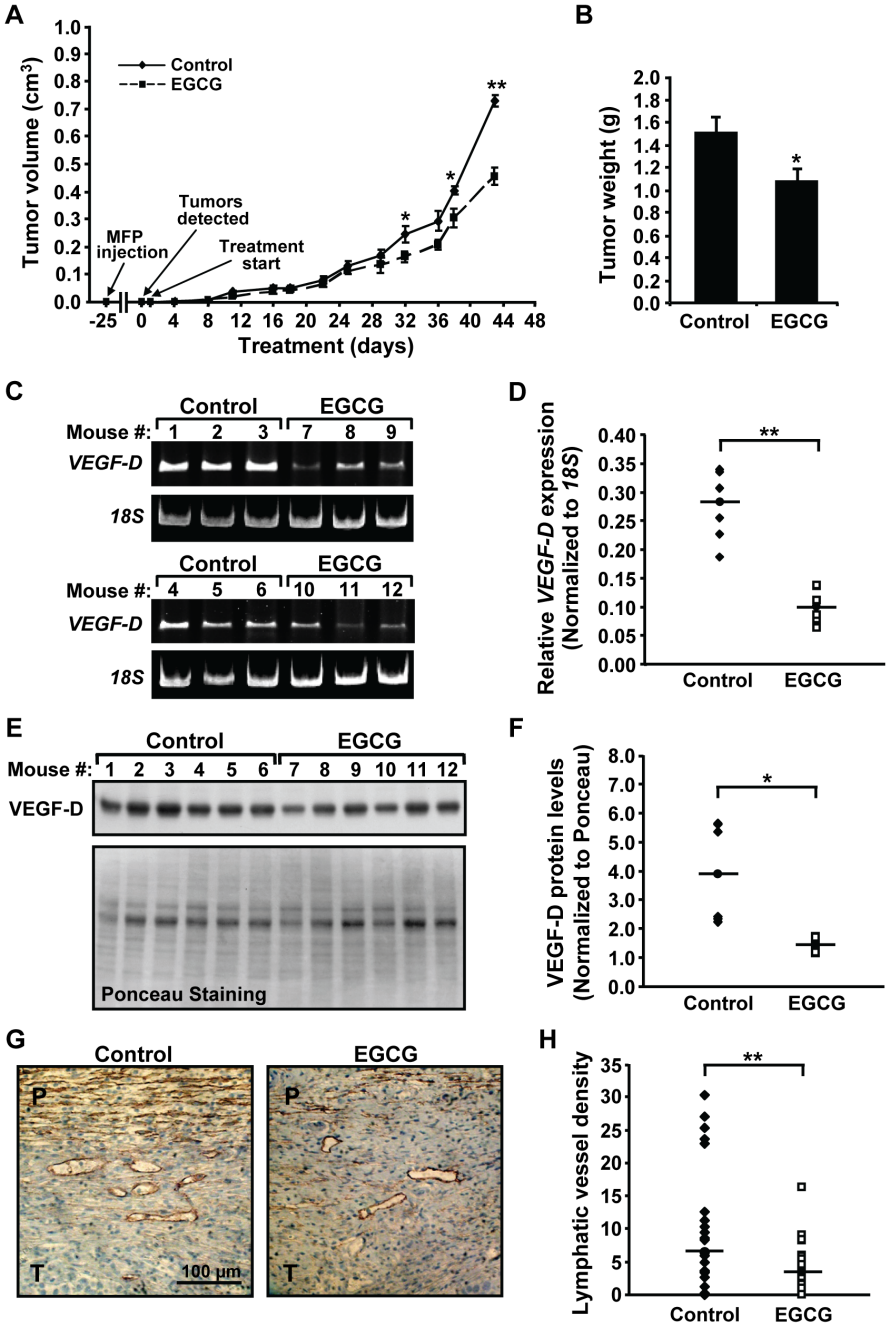
EGCG treatment inhibits growth and lymphangiogenic potential of tumors derived from ALDH-positive cells in mice. (A) Female NOD/SCID mice were implanted with SUM-149 ALDH-positive cells in the 4^th^ inguinal mammary fat pad (MFP injection) and separated into two groups (n = 6/group) once tumors became palpable (Tumors detected). Mice were treated by intraperitoneal injection with 16.5 mg/kg EGCG or Control 1X PBS (Treatment start). Tumor size was measured with calipers twice a week and tumor volumes calculated. Values shown are averages per group ± SE. *, p-value <0.05; **, p-value <0.005. (B) Following sacrifice, tumors were dissected out and weighed. Values shown are averages per group ± SE. *, p-value <0.05. (C–D) Tumor RNA was isolated and analyzed by RT-PCR using primers specific for human *VEGF-D* and for *18S* rRNA (C). Levels of h*VEGF-D* normalized to *18S* rRNA for each mouse per group are presented. Solid line represents median of the group (D). **, p-value <0.005. (E–F) Tumor proteins were extracted and separated by gel electrophoresis. Proteins were stained with Ponceau red and subjected to Western blotting for VEGF-D (E). Values of VEGF-D normalized to total Ponceau staining per sample are presented for each mouse per group. Solid line represents median of the group (F). *, p-value <0.05. (G–H) Tumor sections from Control or EGCG-treated mice were stained with an anti-podoplanin antibody. Representative images at 400× are shown (G). P-peritumoral area; T-tumor. Immunostained sections were analyzed and lymphatic vessel density quantified as described in Materials and Methods (H). Lymphatic vessel density for the Control and EGCG-treated groups is given as the average number of vessels in 3 hot spots in each of 4 podoplanin stained tumor sections per mouse. Solid line represents median of the group. **, p-value <0.005.

To investigate the effects of EGCG on growth of pre-established IBC tumors, ALDH-positive SUM-149 cells were injected into the mammary fat pad of female mice. After 25 days, palpable tumors were detected in all of the mice. Treatment was initiated the next day (day 1) with 16.5 mg/kg EGCG or control PBS 5 days a week. A statistically significant decrease in tumor size was detected in EGCG *vs* control populations at day 32 (Fig. 7A). As we noted a loss of significance at day 36 following a weekend hiatus in treatment, suggesting reversible cytostatic effects of the polyphenol at the dose used, EGCG was administered daily during the last week of the experiment. When tumors in control animals approached a volume of 1 cm^3^, the experiment was concluded. A significant difference in tumor volume was re-established such that a 37.7±4.4% decrease was seen in the EGCG-treated *vs* control groups. Consistently, tumor weight was significantly decreased by 28.6±6.5% in EGCG-treated animals (Fig. 7B). ALDH-positive SUM-149 cells gave rise to high grade invasive carcinomas characterized by anaplastic cells with high mitotic rates and areas of significant necrosis, as judged by H&E staining [34] (data not shown). RT-PCR and immunoblot analyses revealed substantial decreases in levels of human *VEGF-D* RNA (Fig. 7C and 7D) and VEGF-D protein (Fig. 7E and 7F) within tumor tissues of EGCG-treated *vs* control animals. Consistently, immunohistochemistry of tumor sections with an antibody against the lymphatic endothelial cell marker Podoplanin showed a significant decrease in lymphatic vessel density at the periphery of tumors isolated from EGCG-treated mice (Fig. 7G and 7H). Thus, EGCG inhibits the growth and lymphangiogenic capacity of tumors derived from ALDH-positive stem-like SUM-149 cells in a mouse xenograft model.

## Discussion

Here, EGCG is shown for the first time to inhibit the growth and lymphangiogenic potential of pre-existing tumors derived from SUM-149 IBC ALDH-positive stem-like cells in an orthotopic mouse model. Consistently, treatment of SUM-149 IBC stem-like cells or total populations of SUM-149 and SUM-190 IBC cells in culture with the green tea polyphenol caused a dose-dependent inhibition of cell growth and transformed phenotype at lower concentrations and induced apoptosis at higher doses. EGCG treatment also decreased VEGF-D expression in tumors, SUM-149 stem-like cells and in the total populations of SUM-149 and SUM-190 cells, consistent with observed suppression of the ability of the SUM-149 and SUM-190 IBC cells to stimulate *in vitro* lymphatic endothelial cell migration and tube formation. Furthermore, EGCG robustly inhibited formation of tumorspheres by SUM-149 cells, suggesting this polyphenol can reduce the capacity for self-renewal. Regular green tea consumption prior to breast cancer diagnosis has been associated with decreased subsequent risk of recurrence [20]. Our findings suggest that repression of the cancer stem-like cell compartment by green tea polyphenols could play an important role. Given the poor prognosis of IBC patients and our findings demonstrating pleiotropic inhibitory effects of EGCG on this aggressive form of breast cancer, the use of EGCG or green tea in combination with standard treatment modalities for IBC patients warrants further exploration.

Several recent clinical trials for prostate cancer and lymphocytic leukemia using various green tea polyphenol extracts have shown efficacy with low toxicity. Short-term daily supplementation with Polyphenon E, a decaffeinated green tea polyphenol preparation (containing 800 mg of EGCG), in prostate cancer patients during the time between prostate biopsy and radical prostatectomy decreased cancer associated markers HGF, VEGF-A and PSA [35]. Only 3% of patients with high grade prostatic intraepithelial neoplasia given capsules of green tea extracts for one year progressed to prostate cancer compared to 30% in the placebo group [36]. A two-year follow-up demonstrated that this was a long-lasting effect [37]. Polyphenon E with daily EGCG doses ranging from 800 to 4000 mg for 6 months, corresponding to a maximum dose of ∼53 mg/kg for women, to previously untreated chronic lymphocytic leukemia patients with Rai stage 0 to II disease resulted in a significant reduction in absolute lymphocyte count and lymphadenopathy [38]. Polyphenon E and EGCG in ointment or capsules were protective in patients with human papilloma virus-infected cervical lesions [39]. Notably, the EGCG dose used in mice in the present study (16.5 mg/kg) was comparable to the well-tolerated doses given to cancer patients.

The human ALDH superfamily of NAD(P)+-dependent enzymes promotes oxidation of toxic aldehydes into weaker carboxylic acids [40]. High expression of different ALDH family members or total ALDH enzymatic activity are now used to isolate cancer stem-like cells from a variety of solid tumors [40]. ALDH1 expression in particular has been associated with resistance to therapy and a tendency towards disease relapse in breast cancer patients [41], [42]. Previous studies have characterized SUM-149 cells with high ALDH enzymatic activity as an aggressive subpopulation with cancer stem-like cell properties, including self-renewal capacity and high tumorigenicity, and found to mediate invasion *in vitro* and metastasis in mice [16]. Notably in IBC patients, the presence of ALDH1-expressing stem-like cells has been correlated with development of early metastasis and decreased patient survival [16]. EGCG reduced ALDH-positive SUM-149 stem-like cell proliferation *in vitro* and growth of tumors derived from this stem-like cell compartment. This more highly aggressive subpopulation of the SUM-149 cell line was selected in our study to provide a more stringent test of the efficacy of the green tea polyphenol; although, we cannot exclude the possibility that other highly aggressive subpopulations exist within SUM-149 cultures that may not be affected by EGCG. In previous work green tea polyphenols were found to reduce mammosphere formation and stem-like cells in culture. For example, the increase in mammospheres and percent ALDH-positive cells within these structures resulting from carcinogen treatment of untransformed MCF10A cells was blocked by EGCG [43]. Similarly, EGCG derivatives reduced the percent CD44^+^/CD24^−^ cancer stem-like cells in MDA-MB-231 cell cultures and their ability to form mammospheres [44]. To our knowledge, this is the first report demonstrating the efficacy of EGCG in an *in vivo* model against tumors derived from stem-like breast cancer cells.

Interestingly, IBC cells were capable of stimulating endothelial cell function through the secretion of soluble factors, as judged by lymphatic endothelial cell migration and tube formation assays. EGCG treatment reduced IBC cell secretion of VEGF-D, a major regulator of tumor associated lymphangiogenesis, and thereby their ability to stimulate endothelial cell function *in vitro*. Van der Auwera and colleagues report no significant difference in lymphatic vessel parameters (vessel number, vessel perimeter and area covered) at intratumoral or peritumoral areas of IBC tumors [45]. Since our tumors had highly necrotic centers, we chose to assess peritumoral lymph vessels as a representative measurement. EGCG profoundly decreased lymphatic vessel recruitment toward the tumor periphery in mice. Our studies implicate VEGF-D in these paracrine interactions between IBC cells and endothelial cells in the tumor microenvironment. Immunodepletion of VEGF-D from IBC cell media resulted in decreased stimulation of lymphatic endothelial cell migration and tube formation. *VEGFD* ^−/−^ mice showed decreased peritumoral lymphangiogenesis and lymph node metastasis in an orthotopic pancreatic tumor model [46]. Inhibition of VEGF-D in a mammary fat pad xenograft model with a specific antibody prevented lymphangiogenesis and metastasis [14]. In patients with invasive breast cancer, VEGF-D expression in tumor cells correlated with increased lymphatic vessel density, lymph node metastasis and decreased disease-free survival [47], [48]. *VEGF-D* expression is significantly elevated in highly lymphangiogenic IBC tumors compared to non-IBC cancers [12] and similarly in the ALDH-positive SUM-149 IBC stem-like cells. ALDH-positive stem cells have been reported to promote angiogenesis [49] and our observation of increased *VEGF-D* expression are consistent with a role in lymphangiogenesis. EGCG reduced the total levels *VEGF-D* mRNA and VEGF-D protein in tumors derived from ALDH-positive SUM-149 cells, which likely contributed to the decreased lymphatic vessel recruitment at the tumor periphery.

The ability of EGCG to repress a wider array of factors and have more profound phenotypic effects on SUM-149 cells compared to SUM-190 cells may be a result of differences in molecular subtypes, cellular heterogeneity or intrinsic signaling pathways between the two IBC tumors from which they were derived. SUM-149 cells have a triple negative hormone receptor status and are more highly proliferative compared to SUM-190 cells, which are ER-, PR- and Her-2 amplified. Importantly, the triple-negative subtype of IBC has been associated with decreased overall survival and increased rate of locoregional relapse [50]. Thus, our results showing more profound effects on the SUM-149 cell line, suggest the use of EGCG in combination with standard treatments for the triple-negative subtype of IBC may be particularly effective.

### Conclusions

Here, we demonstrate for the first time that the green tea polyphenol EGCG has multiple inhibitory effects on highly malignant IBC cells. EGCG treatment in culture reduces IBC cell proliferation, invasive phenotype and ability to promote lymphangiogenesis at low doses and survival at higher doses. Notably, EGCG decreases growth and lymphangiogenic potential of pre-existing tumors derived from ALDH-positive stem-like cells, which have been associated with poor prognosis of IBC patients.

## Supporting Information

Figure S1 **VEGF-D antibody used in immunodepletion experiments fails to recognize VEGF-A or VEGF-C.** Conditioned concentrated medium from untreated SUM-149 or SUM-190 cells was immunodepleted using a rabbit anti-VEGF-D antibody (sc-25784) (α-VD) or control normal rabbit IgG (sc-2027) (Ctrl), both purchased from Santa Cruz Biotechnology. Immunodepleted media were used in Western blot analysis with mouse monoclonal antibodies VEGF-A (MAB293) and VEGF-C (MAB752) [R&D Systems]. Alternatively, the media were assessed for VEGF-D (Fig. 5B). For each cell line, a representative of two independent experiments is shown. Values given below are averages relative to control sample set to 1± SE from two independent experiments, except for VEGF-A in SUM-149 cells, which is from a single experiment as VEGF-A was below detectable levels in the repeat experiment.
(PPT)

Materials S1 **Reverse transcription (RT)-PCR conditions and primer sets.**
(DOC)
