# Supplementary material for: Epigallocatechin-3-Gallate Inhibits Stem-Like Inflammatory Breast Cancer Cells
Source: PLoS One. 2013 Sep 11;8(9):e73464. doi: 10.1371/journal.pone.0073464 (PMC3770659; doi:10.1371/journal.pone.0073464)
Supplement: Figure S1 — VEGF-D antibody used in immunodepletion experiments fails to recognize VEGF-A or VEGF-C. Conditioned concentrated medium from untreated SUM-149 or SUM-190 cells was immunodepleted using a rabbit anti-VEGF-D antibody (sc-25784) (α-VD) or control normal rabbit IgG (sc-2027) (Ctrl), both purchased from Santa Cruz Biotechnology. Immunodepleted media were used in Western blot analysis with mouse monoclonal antibodies VEGF-A (MAB293) and VEGF-C (MAB752) [R&D Systems]. Alternatively, the media were assessed for VEGF-D (Fig. 5B). For each cell line, a representative of two independent experiments is shown. Values given below are averages relative to control sample set to 1± SE from two independent experiments, except for VEGF-A in SUM-149 cells, which is from a single experiment as VEGF-A was below detectable levels in the repeat experiment. (PPT) [file pone.0073464.s001.ppt]

## Slide 1
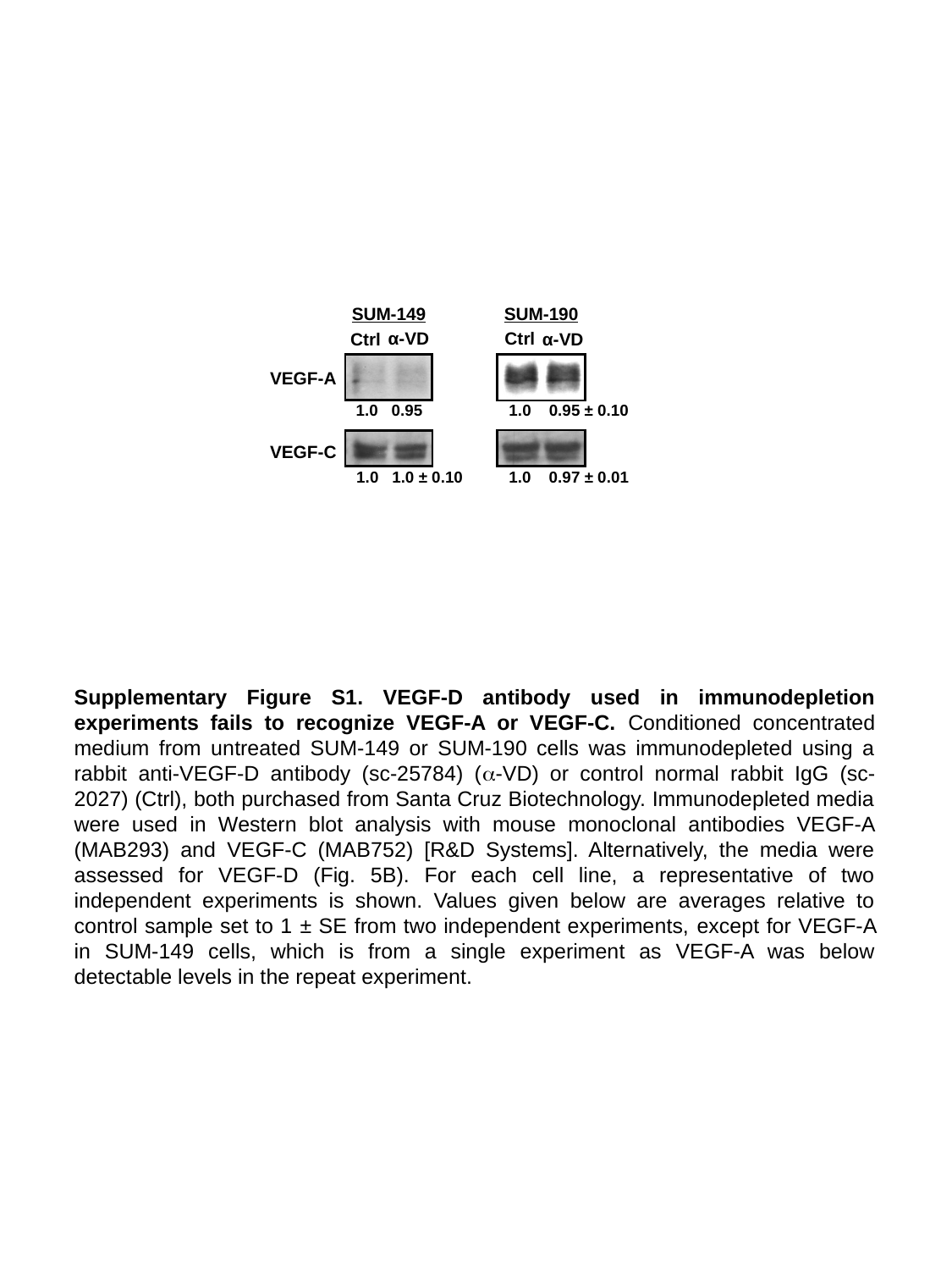

SUM-149
SUM-190
α-VD
Ctrl
Ctrl
α-VD
VEGF-A
 1.0 0.95
1.0 0.95 ± 0.10
VEGF-C
1.0 1.0 ± 0.10
 1.0 0.97 ± 0.01
Supplementary Figure S1. VEGF-D antibody used in immunodepletion experiments fails to recognize VEGF-A or VEGF-C. Conditioned concentrated medium from untreated SUM-149 or SUM-190 cells was immunodepleted using a rabbit anti-VEGF-D antibody (sc-25784) (α-VD) or control normal rabbit IgG (sc-2027) (Ctrl), both purchased from Santa Cruz Biotechnology. Immunodepleted media were used in Western blot analysis with mouse monoclonal antibodies VEGF-A (MAB293) and VEGF-C (MAB752) [R&D Systems]. Alternatively, the media were assessed for VEGF-D (Fig. 5B). For each cell line, a representative of two independent experiments is shown. Values given below are averages relative to control sample set to 1 ± SE from two independent experiments, except for VEGF-A in SUM-149 cells, which is from a single experiment as VEGF-A was below detectable levels in the repeat experiment.
