# Supplementary material for: Epigallocatechin-3-Gallate Inhibits Stem-Like Inflammatory Breast Cancer Cells
Source: PLoS One. 2013 Sep 11;8(9):e73464. doi: 10.1371/journal.pone.0073464 (PMC3770659; doi:10.1371/journal.pone.0073464)
Supplement: Materials S1 — Reverse transcription (RT)-PCR conditions and primer sets. (DOC) [file pone.0073464.s002.doc]

**Supplementary Materials**

RT-PCR

Reverse transcription (RT)-PCR was performed using the following thermal cycler conditions and primer sets:

*CCND1* sense: ‘AGCGCTGTTTTTGTTGTGTG’

*CCND1* antisense: ‘CCTTCCGGTGTGAAACATCT’

95°C for 30 sec, 60°C for 30 sec and 72°C for 45 sec; 24 cycles

*RHOC* sense: ‘ATGGCTGCAATCCGAAAG’

*RHOC* antisense: ‘GATCTCAGAGAATGGGACAGC’

95°C for 30 sec, 60°C for 30 sec and 72°C for 45 sec; 23 cycles

*FN1* sense: ‘GTGCCTGGGCAACGGA’

*FN1* antisense: ‘CCCGACCCTGACCGAAG’

95°C for 30 sec, 55°C for 30 sec and 72°C for 45 sec; 18 and 21 cycles for SUM-149 and SUM-190, respectively

*CDH1* sense: ‘CAGCACGTACACAGCCCTAA’

*CDH1* antisense: ‘ACCTGAGGCTTTGGATTCCT’

95°C for 30 sec, 56.8°C for 30 sec and 72°C for 45 sec; 22 cycles

*VIM* sense: ‘GCTTCAACGGCAAAGTTCTC’

*VIM* antisense: ‘CCTTGAACGCAAAGTGGAAT’

95°C for 30 sec, 55°C for 30 sec and 72°C for 45 sec; 27 and 30 cycles for SUM-149 and SUM-190, respectively

*BCL-XL* sense: ‘TTCAGTGACCTGACATCCCA’

*BCL-XL* antisense: ‘TCCACAAAAGTATCCCAGCC’

95°C for 30 sec, 60°C for 30 sec and 72°C for 45 sec; 24 cycles

*VEGF-A* sense: ‘GCAGAATCATCACGAAGTGG’

*VEGF-A* antisense: ‘GCATGGTGATGTTGGACTCC’

95°C for 30 sec, 60°C for 30 sec and 72°C for 45 sec; 24 cycles

*VEGF-B* sense: ‘CCTTGACTGTGGAGCTCATG’

*VEGF-B* antisense: ‘TGTCTGGCTTCACAGCACTG’

95°C for 30 sec, 60°C for 30 sec and 72°C for 45 sec; 22 and 24 cycles for SUM-149 and SUM-190, respectively

*VEGF-C* sense: ‘AGACTCAATGCATGCCACG’

*VEGF-C* antisense: ‘TTGAGTCATCTCCAGCATCC’

95°C for 30 sec, 60°C for 30 sec and 72°C for 45 sec; 22 and 24 cycles for SUM-149 and SUM-190, respectively

*VEGF-D* sense: ‘GCTGTTGCAATGAAGAGAGC’

*VEGF-D* antisense: ‘TCTTCTGTTCCAGCAAGTGG’

95°C for 30 sec, 60°C for 30 sec and 72°C for 45 sec; 33 cycles

*VEGF-D* sense: ‘TCCAGAGTTGGGTTCCAGCTTTCTG’ (Human specific)

*VEGF-D* antisense: ‘TCTCTCCAATGTATGCCGCAGGT’ (Human specific)

95°C for 30 sec, 60°C for 30 sec and 72°C for 45 sec; 30 cycles

*NANOG* sense: ‘AAGGTCCCGGTCAAGAAACAG’

*NANOG* antisense: ‘CTTCTGCGTCACACCATTGC’

95°C for 30 sec, 62°C for 30 sec and 72°C for 45 sec; 24 cycles

*STELLA* sense: ‘TTAATCCAACCTACATCCCAGGG’

*STELLA* antisense: ‘AGGGGAAACAGATTCGCTACTA’

95°C for 30 sec, 62°C for 30 sec and 72°C for 45 sec; 26 cycles

*GAPDH* sense: ‘TCACCATCTTCCAGGAG’

*GAPDH* antisense: ‘GCTTCACCACCTTCTTG’

95°C for 30 sec, 55°C for 30 sec and 72°C for 45 sec; 18 cycles

*18S* sense: ‘TCAACTTTCGATGGTAGTCGCCGT’

*18S* antisense: ‘TTGCCCTCCAATGGATCCTCGTTA’

95°C for 30 sec, 60°C for 30 sec and 72°C for 45 sec; 27 cycles
